# Mifepristone Overcomes Tumor Resistance to Temozolomide Associated with DNA Damage Repair and Apoptosis in an Orthotopic Model of Glioblastoma

**DOI:** 10.3390/cancers11010016

**Published:** 2018-12-22

**Authors:** Monserrat Llaguno-Munive, Mario Romero-Piña, Janeth Serrano-Bello, Luis A. Medina, Norma Uribe-Uribe, Ana Maria Salazar, Mauricio Rodríguez-Dorantes, Patricia Garcia-Lopez

**Affiliations:** 1 Laboratorio de Farmacología, Subdirección de Investigación Básica, Instituto Nacional de Cancerología, Ciudad de México 14080, Mexico; muniv1250@hotmail.com (M.L.-M.); esau1708@gmail.com (M.R.-P.); 2 Posgrado en Ciencias Biomédicas, Universidad Nacional Autónoma de México (UNAM), Ciudad de México 04510, Mexico; 3 Facultad de Odontología, Universidad Nacional Autónoma de México (UNAM), Ciudad de México 04510, Mexico; janserbe@hotmail.com; 4 Instituto de Física, Universidad Nacional Autónoma de México (UNAM), Unidad de Investigación Biomédica en Cáncer INCan-UNAM, Ciudad de México, 14080, Mexico; medina@fisica.unam.mx; 5 Instituto Nacional de Ciencias Médicas y de la Nutrición Salvador Zubirán, Ciudad de México 14080, Mexico; nofeliauribe@yahoo.com.mx; 6 Instituto de Investigaciones Biomédicas, Universidad Nacional Autónoma de México (UNAM), Ciudad de México 04510, Mexico; anamsm@biomedicas.unam.mx; 7 Instituto de Medicina Genómica, Ciudad de México 14080, Mexico; mrodriguez@inmegen.gob.mx

**Keywords:** glioblastoma, temozolomide, mifepristone, MGMT, drug resistance, apoptosis

## Abstract

The standard treatment for glioblastoma multiforme (GBM) is surgery followed by chemo/radiotherapy. A major limitation on patient improvement is the high resistance of tumors to drug treatment, likely responsible for their subsequent recurrence and rapid progression. Therefore, alternatives to the standard therapy are necessary. The aim of the present study was to evaluate whether mifepristone, an antihormonal agent, has a synergistic effect with temozolomide (used in standard therapy for gliomas). Whereas the mechanism of temozolomide involves damage to tumor DNA leading to apoptosis, tumor resistance is associated with DNA damage repair through the O^6^-methylguanine-DNA-methyltransferase (MGMT) enzyme. Temozolomide/mifepristone treatment, herein examined in Wistar rats after orthotopically implanting C6 glioma cells, markedly reduced proliferation. This was evidenced by a decreased level of the following parameters: a proliferation marker (Ki-67), a tumor growth marker (^18^F-fluorothymidine uptake, determined by PET/CT images), and the MGMT enzyme. Increased apoptosis was detected by the relative expression of related proteins, (e.g. Bcl-2 (B-cell lymphoma 2), Bax (bcl-2-like protein 4) and caspase-3). Thus, greater apoptosis of tumor cells caused by their diminished capacity to repair DNA probably contributed significantly to the enhanced activity of temozolomide. The results suggest that mifepristone could possibly act as a chemo-sensitizing agent for temozolomide during chemotherapy for GBM.

## 1. Introduction

Glioblastoma multiforme (GBM), the most common tumor of the central nervous system, is a highly aggressive cancer with a prognosis of 14.6 months median survival upon diagnosis [1]. Agents for GBM therapy encounter obstacles nonexistent in the treatment of non-neurological cancers. For instance, the blood-brain barrier restricts the passage of many chemotherapeutic agents due to certain well-known characteristics, such as the presence of tight junctions between cells and the lack of fenestrae [2,3].

The current treatment for GBM consists of surgery followed by radiotherapy and chemotherapy with temozolomide. Unfortunately, the great tumor resistance to this drug limits its effectiveness and often leads to recurrence and rapid tumor progression [4]. Subsequent to the oral administration of temozolomide, its spontaneous breakdown at physiological pH produces monomethyltriazene 5-(3-methyltriazen-1-yl)-imidazole-4-carboxamide (MTIC), which in turn reacts with water to liberate 5-aminoimidazole-4-carboxamide (AIC) and the highly reactive methyldiazonium cation. The latter methylates DNA purine residues, preferentially O-6 guanine (O^6^-MeG, 6%) in guanine-rich regions [5], but also N-7 guanine (N^7^-MeG, 70%) and N-3 adenine (N^3^-MeA, 9%). Alkylation of the O^6^ site on guanine promotes the insertion of a thymine instead of a cytosine during DNA replication, which can result in cell death [5].

Tumor cells often show chemoresistance to temozolomide, continuing to proliferate after treatment. Drug resistance is due in part to the overexpression of MGMT (O^6^-methylguanine-DNA-methyltransferase), an enzyme involved in the repair of temozolomide-induced DNA damage. There is an inverse relationship between the levels of MGMT expression and the cellular response to temozolomide [6]. Hence, MGMT expression is a relevant prognostic factor for the response to temozolomide therapy, with an elevated expression linked to drug resistance [7].

Clinical and experimental data have established the resistance of GBM to apoptosis. Defects in apoptotic mechanisms foster tumorigenesis and contribute to the resistance to temozolomide, since its cytotoxic activity is exerted in part through the triggering of apoptosis [8]. In recent years, new molecules have been developed to attempt to overcome the problem of tumor resistance to temozolomide, but with little impact on the prognosis and survival of GBM patients [9,10,11]. One strategy for improving GBM treatment is to seek new chemo-sensitizing agents that enhance the effectiveness of temozolomide by increasing the apoptosis of tumor cells through a decrease in the activity of the DNA repair enzyme.

One possibility is mifepristone, which blocks the capacity of progesterone to stimulate the growth, migration and invasion of human astrocytoma cells lines (such as U373, U87 and D54) [12,13]. This synthetic steroid is used as an abortifacient drug because of its anti-progestational and anti-glucocorticoid action. Our group previously demonstrated an important role of mifepristone in chemo-radio-sensitization, describing its synergistic effect with cisplatin and radiotherapy to act against proliferation of cervical cancer cell lines and cervix xenografts [14]. We also studied the addition of mifepristone to temozolomide-based chemotherapy (accompanied by radiation), observing improved antiproliferative activity on a glioma xenograft in the flank of nude mice. The tumor growth rate was slower than that found with radiation alone or temozolomide alone, indicating a chemo- and radio-sensitizing effect [15].

However, the flank tumor model has drawbacks, especially the absence of the environment provided by the normal brain parenchyma and blood brain barrier. Since these factors are known to influence drug delivery to the tumor [16], orthotopic models are preferred. The aim of the present study was to evaluate, with an immunocompetent orthotopic model based on the intracranial implantation of a glioma cell line in rats, whether mifepristone is able to modulate the growth of temozolomide-treated tumors. The possible mechanism of action of mifepristone as a chemo-sensitizing agent was explored by determining the levels of MGMT (a DNA repair enzyme) as well as the expression of proteins as Bcl-2 (B-cell lymphoma 2), Bax (bcl-2-like protein 4) and caspase that participate in the apoptotic pathway.

## 2. Results

### 2.1. Tumor Growth Assessed by Molecular Imaging

**Figure 1:**
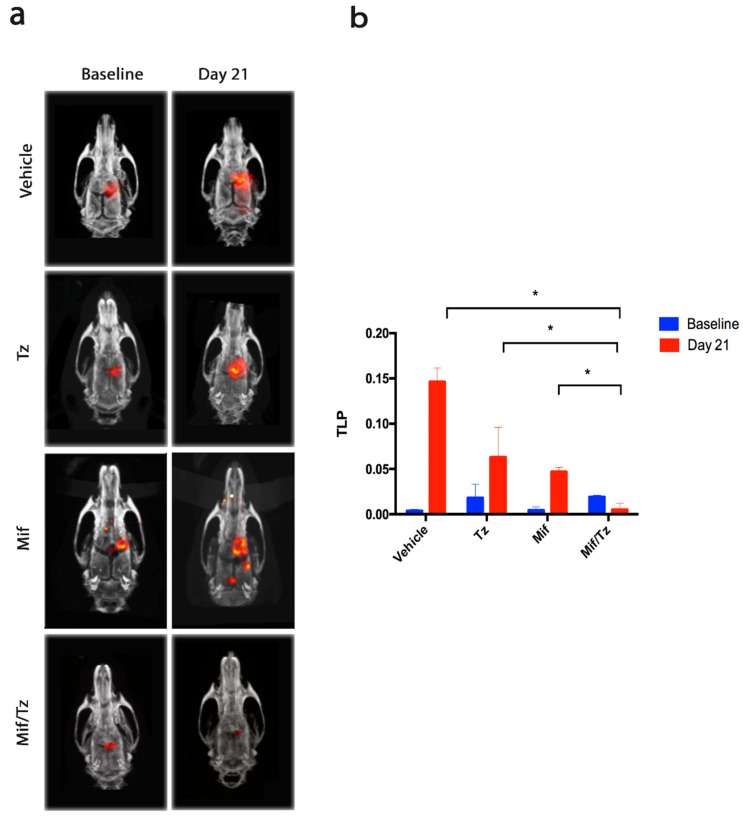
Antitumor activity in the orthotopic rat model of a glioma. (**a**) PET/CT images showing ^18^F-FLT (^18^F-fluorothymidine) tumor uptake for the distinct groups. The images on the left represent the beginning of drug treatment (baseline, two weeks after tumor cell implantation, considered as day 0) and those on the right depict the end of the third week of treatment (day 21). Red reflects the uptake of (^18^F-FLT). (**b**) The proliferative activity of tumors, measured as total lesion proliferation (TLP). Data are expressed as the mean ± SEM of three animals. * A significant difference (*p* < 0.05) between the Mifeprostone/Temozolomide (Mif/Tz) group and the other groups with implantation of glioma cells (the vehicle only, Tz only and Mif only).

Rats were subjected to four treatments: the vehicle only, temozolomide only, mifepristone only, and mifepristone/temozolomide. PET/CT scans were performed at baseline (day 0 of drug treatment, 2 weeks post-implantation) and at the end of the experiment (day 21). In the resulting images (Figure 1a), the presence of red reflects the uptake of ^18^F-fluorothymidine (18F-FLT), a tumor cell growth marker. It is measured as total lesion proliferation (TLP).

After 21 days of treatment, there was an increase in TLP and consequently in the tumor size for the control, temozolomide and mifepristone groups, and a decrease in this parameter for the mifepristone/temozolomide group (Figure 1b). At day 21, both the increase for the vehicle only (control) animals and the decrease for the mifepristone/temozolomide group were significant compared to baseline. Moreover, a significantly lower rate of proliferation was found for the combination treatment than for each of the other three groups (the control, temozolomide only and mifepristone only).

### 2.2. Determination of Body Weight and Overall Survival 

**Figure 2:**
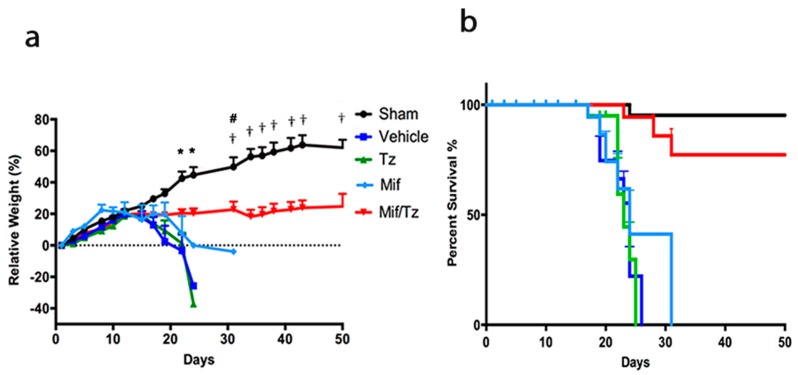
The effect of tumor growth in the orthotopic rat model of a glioma, evaluated by weight loss and overall survival. (**a**) Relative weight of rats in the five groups: sham surgery (without implanting glioma cells) (●), and the implantation of glioma cells followed by each of the treatments: vehicle only (●), temozolomide only (●), mifepristone only (●), and the mifepristone/temozolomide combination (●). Each point represents the mean ± SEM of six animals. * A significant difference (*p* < 0.05) between Mif/Tz and the other groups (Sham, Control, Tz only and Mif only) on day 24. # A significant difference (*p* < 0.05) between Mif/Tz and Mif only on day 31. † A significant difference (*p* < 0.05) between the Mif/Tz and the Sham group on day 31. (**b**) Survival analysis of the same groups for up to 50 days after implantation (day 0 = day of surgery).

Tumor growth was also assessed by means of weight loss (Figure 2a). All animals continued to gain weight during the first two weeks post-implantation of tumor cells. Subsequently, the rats treated with the vehicle or temozolomide only rapidly lost weight, evidencing accelerated tumor proliferation.

The rats in the group given mifepristone only displayed a less drastic weight loss. All animals in these three groups survived between 17 and 32 days (Figure 2b). In contrast, the rats receiving mifepristone/temozolomide maintained their weight throughout the study (Figure 2a), and those having no tumor cell implantation (sham operation) gained weight. The latter two groups were still alive when all the rats of the other three groups had died, and most survived to the end of the study (50 days post-implantation) (Figure 2b).

### 2.3. Histological and Immunohistochemical Analysis

**Figure 3:**
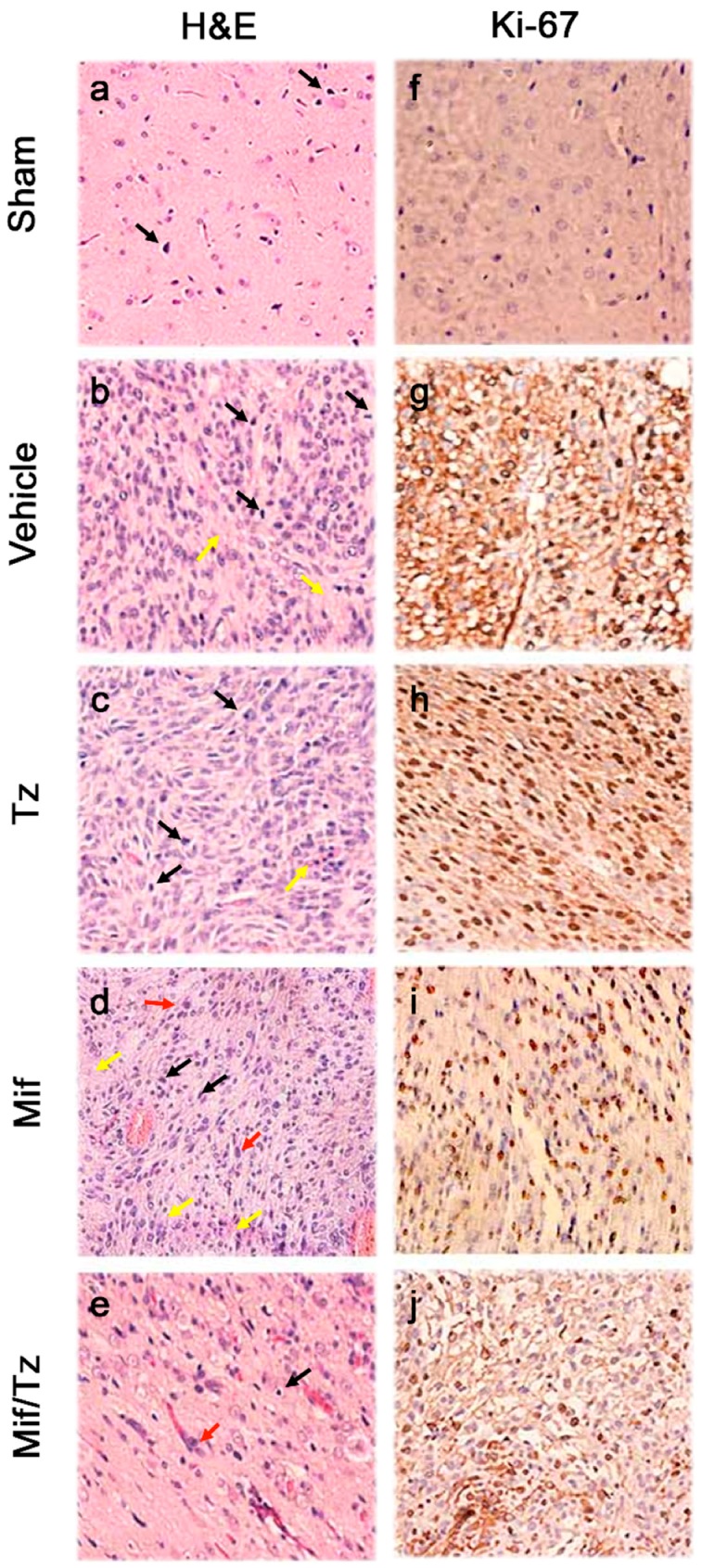
Haematoxylin and eosin (H&E) staining and immunohistochemical analysis of glioma tissue. Sections of tumor tissue were stained with haematoxylin and eosin (**a**–**e**): Mitosis (black arrows), nuclear pleomorphism (red arrows) and necrosis (yellow arrows). Immunostaining of Ki-67 (**f**–**j**), showing few cell nuclei positive to this protein in the tumors of the Mif/Tz group compared to those of the individual treatments (Tz or Mif). The images are representative of three animals per treatment. Magnification 40×.

At the end of the treatments, the brain tissue was processed for histological examination, applying hematoxylin and eosin (H&E) stain to the tissue sections (Figure 3a–e). The tissue of the animals treated with the vehicle only, temozolomide only or mifepristone only showed hypercellularity, mitosis, pleomorphism and necrosis (Figure 3b–d). These characteristics were exhibited to a lesser extent in the rats administered mifepristone/temozolomide (Figure 3e), but with a hypo- versus hypercellular lesion.

Immunohistochemical staining of brain tumor tissue was employed to detect the presence of Ki-67 (Figure 3f–j), a protein expressed by proliferating cells. Compared to the groups receiving the vehicle only, temozolomide alone or mifepristone alone, rats given mifepristone/temozolomide displayed a lower proportion of Ki-67 positive cells in tumors. Indeed, the stained sections from the latter group were similar to those from the sham animals, indicating few cells undergoing proliferation. For animals administered mifepristone only, there was slightly diminished proliferation compared to those in the control and temozolomide only groups. In the mifepristone/temozolomide group, a close correlation can be observed between a lower level of Ki-67 and the significant reduction in tumor growth illustrated in the PET/CT images.

### 2.4. Expression of Apoptotic Proteins

**Figure 4:**
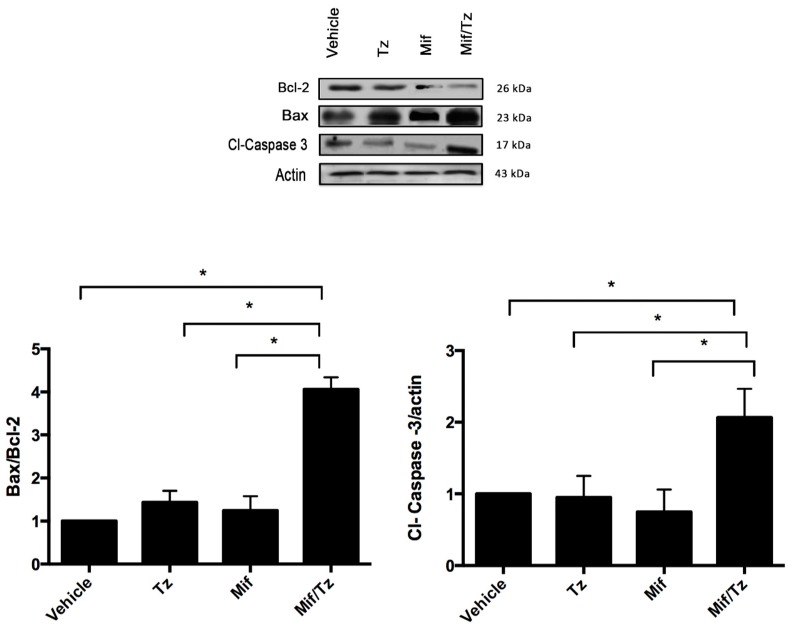
Expression of apoptotic proteins. Representative western blot results and densitometric analysis of Bcl-2, Bax and Cl-Caspase 3. Data are expressed as the mean ± SEM from three independent experiments. * Indicates a significant difference (*p* < 0.05) between Mif/Tz and the other groups with tumor cell implantation (given Tz, Mif or the vehicle).

The combination treatment also affected the expression of apoptotic proteins in tumor cells (Figure 4). Compared to the control group, animals with temozolomide only or mifepristone only displayed a slight increase in the Bax/Bcl-2 ratio, but no change in the expression of cleaved caspase 3. In animals given mifepristone/temozolomide, contrarily, a significant increase was found in the Bax/Bcl-2 ratio and the expression of cleaved caspase 3, revealing a synergy between these two drugs.

### 2.5. Expression of MGMT

**Figure 5:**
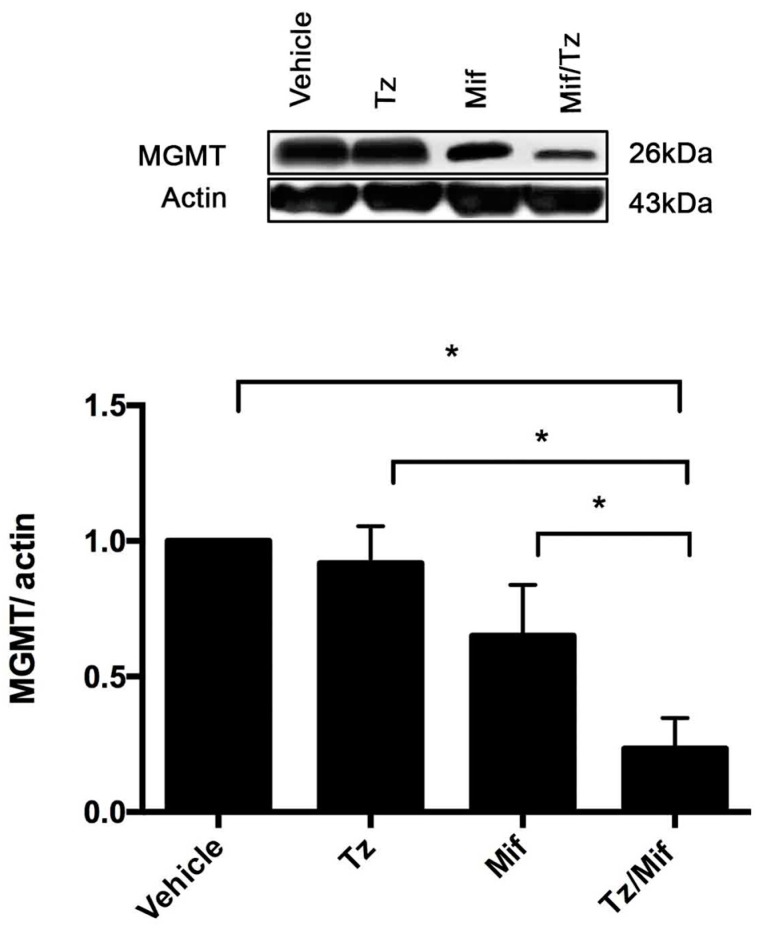
Expression of MGMT (O6-methylguanine-DNA-methyltransferase). Representative western blot results and densitometric analysis of the MGMT protein. Data are expressed as the mean ± SEM of three independent experiments. * A significant difference (*p* < 0.05) between Mif/Tz and the other groups with tumor cell implantation (given Tz, Mif or the vehicle).

MGMT, an enzyme involved in DNA repair, plays a significant role in the resistance of glioma tumors to temozolomide. Western blot data and band intensity showed the protein expression of MGMT to be strikingly downregulated in the mifepristone/temozolomide group and slightly decreased in the mifepristone only group compared to the animals administered temozolomide only (Figure 5).

## 3. Discussion

One of the major problems in the treatment of GBM is the drug resistance of tumors and the repopulation carried out by cells that escape chemotherapy. The standard treatment for this disease, consisting of surgery followed by radiation therapy plus chemotherapy with temozolomide, yields a median survival of only 1–2 years. Therefore, it is essential to find new strategies for treating GBM in order to lengthen patient survival and avoid recurrence.

The current study evaluated whether the combination of mifepristone and temozolomide could give a better outcome than temozolomide only applied to glioma tumors in rats. Mifepristone is known to act as an antagonist of progestins, glucocorticoids and androgens by blocking progesterone receptor (PR), glucocorticoid receptor (GR) and androgen receptor (AR), respectively [17,18]. However, the role of this antihormonal agent as a chemo-sensitizer in GBM has scarcely been explored. Although we previously suggested the potential of mifepristone as a chemo-radio-sensitizer for the standard treatment of a glioma tumor based on a subcutaneous xenograft model of GBM [15], the present intracerebral model more closely resembles the native niche of such a tumor in the central nervous system.

A significant difference in tumor growth was observed between the animals administered mifepristone/temozolomide and those given the vehicle only, temozolomide only or mifepristone only. Compared to the latter three groups, the animals submitted to the combination treatment exhibited significantly reduced tumor growth and greater survival time.

The level of Ki-67 was herein scrutinized due to its expression by proliferating cells in all phases of the active cell cycle and its absence in resting cells (G_0_). Ki-67 was abundant in the tumor cells of the groups given temozolomide only, mifepristone only or the vehicle only, but was scarcely detected in tumor cells of the animals receiving mifepristone/temozolomide. Hence, the mifepristone/ temozolomide combination blocked the proliferation of glioma cells.

The PET/CT scans, which measured ^18^F-FLT uptakes, were in agreement with the Ki-67 results, revealing a lack of tumor cell growth with the combined treatment. ^18^F-FLT displays minimal uptake in normal brain tissue because the expression level of thymidine kinase-1 is very limited in neurons and glia. Thus, ^18^F-FLT/PET may be advantageous for examining tumor recurrence [19,20].

Mifepristone is known to diminish the proliferation of cancer cells of reproductive and non-reproductive origin [21] and to inhibit the in vitro growth of cancer cells derived from the nervous system, breast, prostate, and ovary. It also gives rise to G1-S blockage of the cell cycle through inhibition of cdk2 activity in human ovarian cancer cells [22].

Whereas progesterone is known to generate the proliferation of tumor cells, the progesterone/mifepristone combination has proven to block the proliferative effect. This combination also significantly decreases the tumor area compared to progesterone only administered to rats [23]. Experimental studies have indicated that progesterone is capable of stimulating the infiltration and migration of astrocytes to the rat cortex, exerting its effects through two mechanisms. The first implies an interaction with nuclear PR (nPR), while the second requires the participation of membrane receptors (mPR) [24]. PR expression exists in several types of brain tumors, including meningiomas and gliomas, and increases with histological malignancy [25]. Moreover, mPRs are expressed in GBM cell lines such as U251 and U87 [26]. Upon evaluating the expression of PRs in C6 glioma cells, Su et al. observed no expression of classical nuclear PRs but identified significant levels of mPRs [27].

Although mifepristone plus temozolomide herein diminished tumor growth, its mechanism of action as a chemo-sensitizing agent is unknown. The promotion of apoptosis by mifepristone has been documented in distinct tumor types. Li et al. demonstrated that this drug produces an antiproliferative effect on human SGC-79901 gastric adenocarcinoma cells by downregulating Bcl-XL expression and upregulating caspase 3 activity [28]. Moreover, mifepristone inhibits cell growth by arresting cell cycle progression at the S phase, triggers apoptosis by activating caspase-3, and modulates the genes involved in apoptosis (including BCL2/BAX and FAS/FASLG) in Ishikawa cells [29]. According to Gonzalez-Agüero et al., progesterone significantly increases the growth of U373 and D54 human astrocytoma cells, an outcome blocked by mifepristone in both cases. Additionally, mifepristone administered without progesterone reduced the growth of these two cell lines [12], evidencing a possible antiproliferative effect on gliomas.

Mifepristone/temozolomide treatment presently exerted a strong downregulation of the expression of the antiapoptotic protein Bcl-2 and an upregulation of the expression of two apoptotic proteins (Bax and cl-caspase-3) (Figure 4). This suggests that mifepristone/temozolomide induces apoptosis in glioblastoma tumors in vivo, which could be relevant for improving the effectiveness of standard therapy for GBM.

On the other hand, the epigenetic silencing of MGMT was shown to enhance the response of GBM patients to chemotherapy based on alkylating agents, leading to longer mean survival. The median survival of patients treated with temozolomide is greater when the promoter of MGMT is hypermethylated [30,31]. Whereas most patients are resistant to temozolomide, epigenetic inactivation of MGMT, occurring in approximately 40% of patients, is associated with a better response. [32]. Therefore, the depletion of MGMT is important for increasing tumor sensitivity to temozolomide.

It is still unknown whether mifepristone epigenetically inhibits MGMT. However, some previously described mechanisms could partially account for the current results. Different nuclear transcription factors, including SP1 (protein 1-binding), AP-1 (activator protein), NF-κB (nuclear factor for the polypeptide gene enhancer in B cells) and HIF-1α (hypoxia inducible factor-1α), can also activate the transcription of the MGMT gene [33]. Some of the aforementioned transcription factors may participate in MGMT gene regulation by mifepristone. It has recently been documented that MGMT expression is associated with SP1 expression in glioma cell lines. Since knockdown of SP1 strongly reduced MGMT protein expression, SP1 is one of the main factors regulating MGMT [34]. MGMT expression also depends on p53, a transcription factor that sequesters SP1 and prevents its binding to MGMT [34]. Additionally, there is evidence of mifepristone increasing apoptosis due to p53 activation in several cancer cell lines [35,36]. Hence, the present findings could be partially explained by some of these mechanisms. Further studies are needed to clearly define the mechanisms responsible for the inhibition of MGMT by mifepristone.

Glucocorticoids are frequently used in glioblastoma therapy to address edema. According to some authors, they are involved in eliciting the expression of the MGMT gene, meaning that they could contribute to an elevated MGMT protein level. Biswas et al. and Ueda et al. detected an upregulation of MGMT in glioblastoma cell lines during glucocorticoid treatment [37,38]. Furthermore, Horiguchi et al. found that dexamethasone acts as a positive regulator of hepatic MGMT expression, an effect reversed by concomitant administration of its antagonist mifepristone [39]. The present results are the first, to our knowledge, in relation to the influence of mifepristone on MGMT expression when administered alone. The current mifepristone only treatment was able to decrease the levels of MGMT compared to the group given the vehicle only and temozolomide only. This could account for the enhancement of sensitivity of tumor cells to temozolomide, observed in the group receiving the mifepristone/temozolomide combination (Figure 5).

A correlation existed between a higher MGMT expression and greater resistance of the tumor to treatment (the latter reflected in the lower survival of the animals). Whereas the rats administered temozolomide only did not survive more than 24 days, those with the mifepristone/temozolomide combination showed a remarkable increase in mean survival. About 75% of the animals receiving the latter treatment survived to the end of the experiment (50 days).

**Figure 6:**
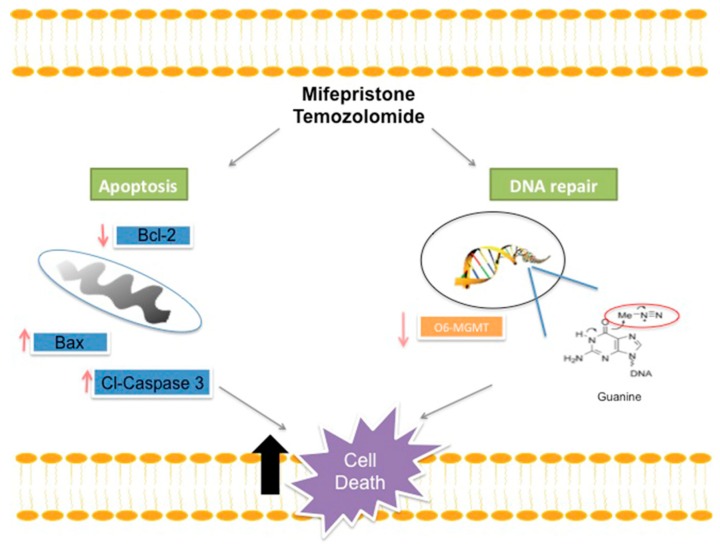
Schematic portrayal of the possible mechanisms of treatment with mifepristone/temozolomide. This combination decreased the level of anti-apoptotic protein Bcl-2 and increased the levels of pro-apoptotic proteins Bax and cl-caspase-3, leading to greater cellular apoptosis. As described elsewhere, temozolomide releases a compound, O^6^-guanine, that causes DNA damage and cell death. However, there are different mechanisms of repair or avoidance employed by tumor cells. For example, resistance to temozolomide treatment is reportedly related to the presence of the MGMT enzyme, which removes the methyl group from O^6^-guanine and thus restores the cellular replication of tumor cells. A lower expression of MGMT was herein detected when administering mifepristone/temozolomide versus temozolomide alone. The combination treatment perhaps allows methylation to occur in the purine bases of DNA at the O^6^ position of guanine without removing the methyl groups, and consequently contributes to an increase in the effect of temozolomide.

A possible mechanism of mifepristone for improving the efficacy of temozolomide is proposed (Figure 6). Drug resistance to temozolomide is associated with DNA damage repair, which impedes apoptosis. Among the numerous mechanisms utilized by glioma cells to resist temozolomide-induced DNA damage, the avoidance of apoptosis is probably one of the most frequently studied. It has been reported that glioma tumors have elevated levels of the anti-apoptotic proteins Bcl-2 and reduced levels of the apoptotic protein Bax, giving rise to a predisposed anti-apoptotic state correlated with resistance to chemotherapy.

Consequently, the inhibition of Bcl-2 through mifepristone treatment, fostering higher levels of Bax and Cl-Caspase, may be an effective strategy for overcoming the resistance of tumors to apoptosis when administering temozolomide. Although new small molecules have been investigated for the inhibition of the Bcl-2 family of proteins in numerous cancer types, their application to GBM has been scarcely explored.

Temozolomide generates numerous DNA adducts (e.g., O^6^-methylguanine) that are regarded as very cytotoxic. Contrarily, MGMT removes alkylating adducts from the O^6^ position of guanine and thus protects glioma cells from this cytotoxicity. A decrease in the levels of MGMT by mifepristone would impede the repair of temozolomide-induced damage, leading to an eventual accumulation of adducts, a reduction of replication and the triggering of cell death.

Mifepristone has been widely studied as an abortifacient and contraceptive drug. According to recent evidence and the current findings, this drug seems to have great potential for cancer treatment. It offers palliative benefits for a wide variety of human cancer types, including patients with glioblastoma (demonstrating that it successfully crosses the blood-brain barrier) [40,41]. Mifepristone has only a mild adverse effect, even when taken daily for up to 13 years [42]. Therefore, long-term administration of mifepristone may be feasible and clinically well-tolerated in combination with temozolomide to treat patients suffering from a glioma. We propose a Phase I clinical trial to test this combination in the near future.

## 4. Materials and Methods

### 4.1. Drugs and Reagents

Mifepristone and temozolomide were obtained from Sigma Chemical Co. (St. Louis, MO, USA). Dulbecco’s modified Eagle’s medium (DMEM), fetal calf serum (FCS), ethylenediaminetetracetic acid (EDTA) and SDS were purchased from Gibco, BRL (Grand Island, NY, USA). High-quality water for the solutions was processed with a Milli-Q Reagent Water System (Continental Water Systems; El Paso, TX, USA). Temozolomide was prepared in DMSO at a final concentration of 4% and mifepristone was reconstituted in polyethylenglycol and saline solution. All standard solutions were stored at −20 °C until use.

### 4.2. Animals

Male Wistar rats, (200–230 g) were supplied by the Instituto Nacional de Ciencias Médicas y Nutrición Salvador Zubiran (INCMNSZ), Mexico City, Mexico. All procedures for the care and handling of the animals were approved by the Ethics Committee of the “Instituto Nacional de Cancerología” (INCan, Mexico City, Mexico), and were in accordance with Mexican Federal Regulations for Animal Experimentation and Care (NOM-062-ZOO-1999, Ministry of Agriculture, Mexico).

### 4.3. Cell Culture

The glioma C6 cell line was acquired from American Type Culture Collection (Rockville, MD, USA). It was routinely maintained as a monolayer in DMEM, supplemented with 5% fetal bovine serum and incubated at 37 °C in 5% CO_2_ atmosphere at high humidity. Cells were harvested with 1mM EDTA.

### 4.4. Tumor Cell Implantation

Posterior to anaesthetization with a combination of tiletamine hydrochloride (10 mg/kg) and acepromazine maleate (0.4 mg/kg) administered s.c., each animal was placed in a stereotactic device for surgery. After fastening the head in the frame, a midline incision was made and bregma was identified. The skull was then drilled at the coordinates of 2.0 mm right from bregma and 6 mm deep (hippocampus), in accordance with the Paxinos and Watson atlas [43].

C6 cells were harvested, washed three times and diluted in DMEM to a concentration of 7.5 × 10^5^ in a volume of 3 μL. Employing an infusion pump and a 27-gauge needle, these cells were slowly implanted at a depth of 6 mm from the dura mater. The injection was made over a 6-min period. Upon closing the scalp, the rat was returned to the animal facility. The rats of the sham group were submitted only to the surgical procedure, without the implantation of tumor cells. Animals were subsequently weighed 3 times/week during the study.

### 4.5. Treatments

At two weeks post-implantation of tumor cells, the animals were divided into four groups (*n* = 6–8): (A) temozolomide only (5 mg/kg/day i.p), (B) mifepristone only (10 mg/kg/day s.c.), (C) mifepristone plus temozolomide (the same doses), and (D) control animals (vehicle only). A fifth group consisted of sham animals (inoculated only with DMEM, in the absence of glioma cells). The drug treatments were administered in three cycles of five consecutive days over a period of three weeks. The antitumor activity of temozolomide is schedule-dependent, with multiple administrations being more effective than a single treatment. In clinical practice, the recommended dose of temozolomide is 75–200 mg/m^2^ given orally for 5 consecutive days every 28-day cycle (5/28 d) [44,45].

### 4.6. Tumor Growth Assessed by Molecular Imaging

Brain tumor growth was measured with a microPET/CT scanner (Albira ARS, Oncovision Valencia, Spain). PET/CT images were acquired at 2 weeks post-implantation (day 0 of drug treatment) and subsequently on a weekly basis up to the time of euthanization. 300 μCi of 18 F-FLT was injected into the caudal vein of rats under O_2_/isoflurane anesthesia (1–3% isoflurane in 100% oxygen). The images were acquired at 40–60 min post-injection.

### 4.7. Determination of Body Weight and Overall Survival

Tumor growth was also evaluated by monitoring weight loss during treatments and recording the global survival of animals throughout the study, which lasted 50 days.

### 4.8. Histology and Immunohistochemistry

At the end of the experiment, all rats were euthanized and perfused with saline solution followed by 4% paraformaldehyde. Brains were removed and immersed in 4% paraformaldehyde for 2 weeks. The brain tissue was embedded in paraffin and sliced into sections (2 mm thick) on the coronal plane. The tissue slices were stained using the H&E method, and cell proliferation examined according to the level of the Ki-67 protein. A section of rat spleen was employed as a positive control.

### 4.9. Western Blot Analysis

Tissue samples were homogenized with a lysis buffer containing protease inhibitors. Proteins were obtained by centrifugation at 10,000 g and 4 °C, separated on 10% polyacrylamide gel and quantified by bicinchoninic acid assay (BCA). Colored markers (Bio-Rad, Hercules, CA, USA) were included to establish size. Proteins were then electrophoretically transferred from the gel onto PVDF membranes (Amersham, Buckinghamshire, UK), which were blocked with 5% non-fat dry milk at room temperature for 2 h. Membranes were incubated overnight at 4 °C with antibodies against MGMT (sc-166528, 1:500), Bcl-2 (sc-7382, 1:1000), Bax (sc-20067, 1:1000), actin (sc-69879, 1:1000; Santa Cruz Biotechnology, Dallas, TX, USA) and cleaved caspase 3 (9661, 1:1000; Cell Signaling Technology, MAB230, R&D Systems, Minneapolis, MN, USA). After washing, the membranes were incubated with anti-mouse or anti-rabbit secondary antibodies, IgG-HRP (1:1500 and 1:3000, respectively; Santa Cruz Biotechnology) for 1 h.

Blots were developed with a chemiluminescent substrate (ECL Prime). Chemiluminescent bands were developed on Kodak autoradiography film in a darkroom and their densities were measured on Image Studio software (version, 5.2 Li-cor, Lincoln, NE, USA). In each figure, representative blot images were selected from the same gel.

### 4.10. Statistical Analysis

Values are expressed as the mean ± SEM (standard error of the mean). Statistical analysis was performed with one-way analysis of variance (ANOVA) on SPSS Base 20.0 software (SPSS Inc., Chicago, IL, USA). Differences were scrutinized with multiple comparisons between groups. When necessary, the comparison of means was Bonferroni adjusted. In all cases, a value of *p* < 0.05 was considered significant.

## 5. Conclusions

The present results indicate that mifepristone was capable of enhancing the inhibitory effect of temozolomide on the proliferation of glioma tumors in an orthotopic rat model of glioblastoma. Hence, this antihormonal drug could possibly be beneficial as a sensitizer for temozolomide in standard therapy for GBM. Since standard GBM treatment is of limited effectiveness, evidenced by low patient survival, the current findings are encouraging and suggest the importance of a Phase I clinical study to further explore the combination of mifepristone and temozolomide.
